# Patient recruitment into clinical studies of solid malignancies during the COVID-19 pandemic in a tertiary cancer center

**DOI:** 10.1016/j.neo.2023.100946

**Published:** 2023-10-27

**Authors:** Jens von der Grün, Maiwand Ahmadsei, Isabel Breyer, Christian Britschgi, Daniel Eberli, Thomas Hermanns, Joanna Mangana, Henrik Petrowsky, Egle Ramelyte, Patrick Roth, Gabriel Schär, Isabelle Opitz, Michael Weller, Andreas Wicki, Isabell Witzel, Panagiotis Balermpas, Matthias Guckenberger

**Affiliations:** a Department of Radiation Oncology, University Hospital Zurich and University of Zurich, Zurich, Switzerland; b Comprehensive Cancer Center Zurich, University Hospital Zurich and University of Zurich, Zurich, Switzerland; c Department of Gynecology, University Hospital Zurich and University of Zurich, Zurich, Switzerland; d Department of Department of Medical Oncology and Hematology, University Hospital Zurich and University of Zurich, Zurich, Switzerland; e Department of Urology, University Hospital Zurich and University of Zurich, Zurich, Switzerland; f Department of Dermatology, University Hospital Zurich and University of Zurich, Zurich, Switzerland; g Department of Surgery and Transplantation, University Hospital Zurich and University of Zurich, Zurich, Switzerland; h Department of Neurology & Brain Tumor Center, University Hospital Zurich and University of Zurich, Zurich, Switzerland; i Department of Thoracic Surgery, University Hospital Zurich and University of Zurich, Zurich, Switzerland

**Keywords:** COVID-19, Trial recruitment, Oncology, Tertiary cancer center, Solid tumors

## Abstract

**Background and purpose:**

To analyze clinical trial activities and patient recruitment numbers into prospective clinical studies for solid malignancies during the COVID-19 pandemic in a tertiary cancer center.

**Materials and methods:**

Patient recruitment numbers in prospective clinical studies of solid malignancies were retrospectively analyzed for the years 2019 – 2021 at the Comprehensive Cancer Center Zurich (CCCZ). Changes in recruitment numbers were tested for association with organ-specific subunits, as well as organizational and treatment-related trial characteristics. To assess differences between categorical variables, Chi-squared test was used. For uni- and multivariate analysis, Cox proportional hazards were calculated.

**Results:**

In 2019, there were a total of 107 studies (registry trials, clinical phase I-III trials, and translational studies) recruiting 304 patients at the CCCZ. During the COVID-19 pandemic in 2020 and 2021, there were 120 and 125 active trials with a total recruitment of 355 and 666 patients, respectively. No significant differences between the subunits and study characteristics in changes of patient recruitment in clinical phase I-III trials were identified when the year prior to the COVID-19 pandemic (2019) was compared to the first year of the pandemic (2020) and to 2020-2021.

**Conclusions:**

Despite healthcare systems around the world have experienced significant disruption due to the COVID-19 pandemic, data from our tertiary cancer center showed that clinical trial activities were maintained at a high level during the pandemic.

## Highlights

• Assessment of clinical trial activities during the COVID-19 pandemic.
• No differences between the organ specific subunits and study characteristics.
• Data showed that clinical trial activities were maintained during the pandemic.

## Introduction

The COVID-19 pandemic has caused widespread disruption to healthcare systems worldwide, affecting not only the management of patients with COVID-19, but also the care of patients with other medical conditions, including cancer [1], [2], [3], [4]. In particular, the pandemic has posed significant challenges for the conduct of clinical trials, which are critical for advancing cancer research and developing new treatments [5,6].

The COVID-19 pandemic has presented unprecedented barriers to the recruitment of patients for clinical trials, including reduced availability of healthcare resources, travel restrictions, and concerns related to patient safety and exposure to the virus [6,7]. With many trials being delayed or suspended due to challenges in enrolling and retaining participants, the pandemic has also highlighted the need for innovative approaches to clinical trial design and recruitment, particularly for oncology studies [8], [9], [10].

However, general and healthcare-related restrictions during the pandemic varied substantially between the countries and between regions. As indicated by the stringency indexes, Swiss COVID-19 restrictions were more moderate than for example Germany or the United States of America (USA) [11,12]. The less stringent Swiss COVID-19 restrictions resulted in a rather quick recovery of the Swiss health care system after the first pandemic wave in 2020, especially in oncological surgery [13]. However, very little is known about clinical trial activities in the field of oncology, which is affected by local, national, and international regulations.

The purpose of this study was to comprehensively assess clinical trial activities and patient recruitment numbers into clinical studies for solid malignancies during the COVID-19 pandemic in a tertiary cancer center.

## Materials and methods

### Data collection

The organ-specific subunits of the Comprehensive Cancer Center Zürich (CCCZ) retrospectively assessed their clinical trial activity for the years 2019-2021 with regard to the following characteristics: study type and phase, interventional vs. non-interventional study, sponsor, international vs. national, multi-center vs. single-center, locoregional vs. systemic disease, surgery / radiotherapy / systemic therapy as part of the study protocol, and number of enrolled patients per year.

The assembled clinical trial records of the clinical program of the CCCZ were subsequently analyzed to identify the total number of patients recruited in clinical phase I-III, registry trials and translational studies for solid cancers at the University Hospital Zurich (USZ).

### Statistical Analysis and Visualization

Statistical analyzes were performed with SPSS (IBM SPSS Statistics, V29.0, Armonk, NY, USA), RStudio Team (RStudio: Integrated Development for R. RStudio, PBC, Boston, MA), and Excel (Microsoft Excel 2016, Redmond, WA, USA). To assess differences between categorical variables, Chi-squared test was used. For uni- and multivariate analysis, Cox proportional hazards were calculated. To avoid overfitting, a minimum of 10 events per variable was mandatory for each multivariate analysis [14]. Correction for multiple testing was conducted using the Benjamini-Hochberg procedure [15]. *P*-values of <0.05 were considered as significant. For data visualization, Adobe Illustrator (Adobe Inc. Illustrator, V27.3.1, DE, USA) was utilized.

## Results

### COVID-19 development in Switzerland

The official registration of the cumulative COVID-19 case numbers of Switzerland and Liechtenstein started on March 1st, 2020 following the first confirmation of a COVID-19 case in Switzerland on February 25th, 2020. An initial, complete lockdown in Switzerland was established on March 16th, 2020. The lockdown was stepwise relaxed and mainly reduced to social distancing and banning of larger social events between April 27th and June 15th, 2020. During the second and third pandemic waves, an incomplete lockdown was initiated on October 19th, and was further intensified on December 12th, 2020. On December 19th, 2020, SwissMedic approved the first COVID-19 vaccine in Switzerland (Comirnaty, Pfizer/Biontech). Between March 1st and June 26th, 2021, restrictions were lifted stepwise. With regard to the fourth and fifth pandemic waves in September and October 2021, no lockdowns were implemented [16]. A comparison of the Swiss COVID-19 containment policy to those of Germany and the USA are shown in the Supplementary Figs. 1 and 2 [11,12], indicating less stringent COVID-19 restrictions for Switzerland after the first pandemic wave.

**Fig. 1:**
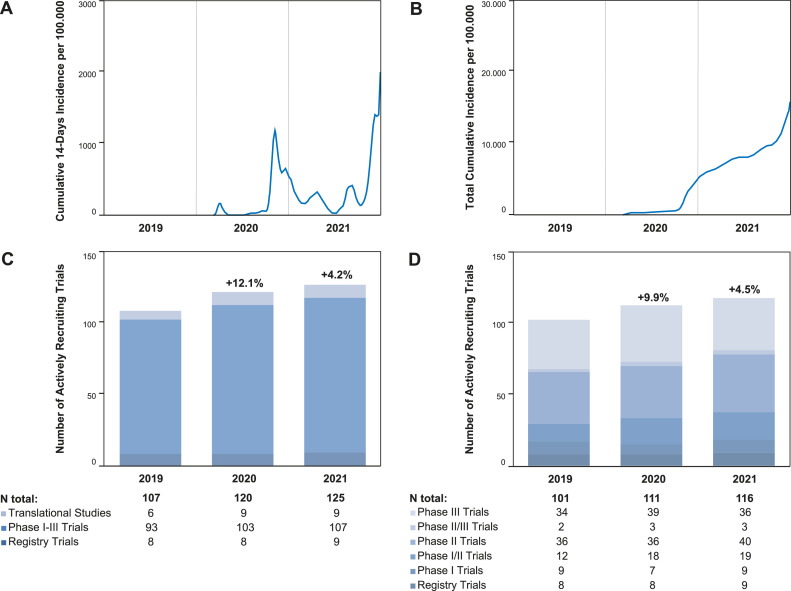
Regional COVID-19 Incidences and Numbers of Recruiting Trials from 2019-2021. A: The graph represents the sum of the last 14 days as the incidence (cases per 100.000 inhabitants) of laboratory-⁠ confirmed COVID-19 cases from March 1st, 2020 until December 31st, 2021 in Switzerland and Liechtenstein (17). B: The graph shows the total cumulative incidence per 100.000 inhabitants of laboratory-⁠ confirmed COVID-19 cases from March 1st, 2020 until December 31st, 2021 in Switzerland and Liechtenstein (17). C: The column chart shows the Number of actively recruiting trials per year at the Comprehensive Cancer Center Zurich. D: The column chart shows the number of actively recruiting clinical phase I-III trials per year at the Comprehensive Cancer Center Zurich.

By the end of 2021, five COVID-19 waves were recorded with a total of 436.134 laboratory-confirmed cases in Switzerland and Liechtenstein [17]. Cumulative incidences are shown in Fig. 1 [17]. By that time, 5.992.658 inhabitants of these two countries had received at least one dose of an anti-Sars-CoV vaccine [17].

### Trials and numbers of patients recruited at the CCCZ in 2019

**Fig. 2:**
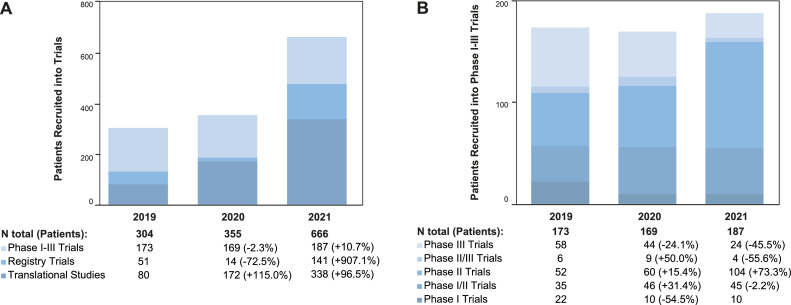
Numbers of Patients recruited in Trials from 2019-2021. A: The column chart shows the number of patients per study type recruited per year. B: The column chart shows the number of patients per study phase recruited per year.

**Fig. 3:**
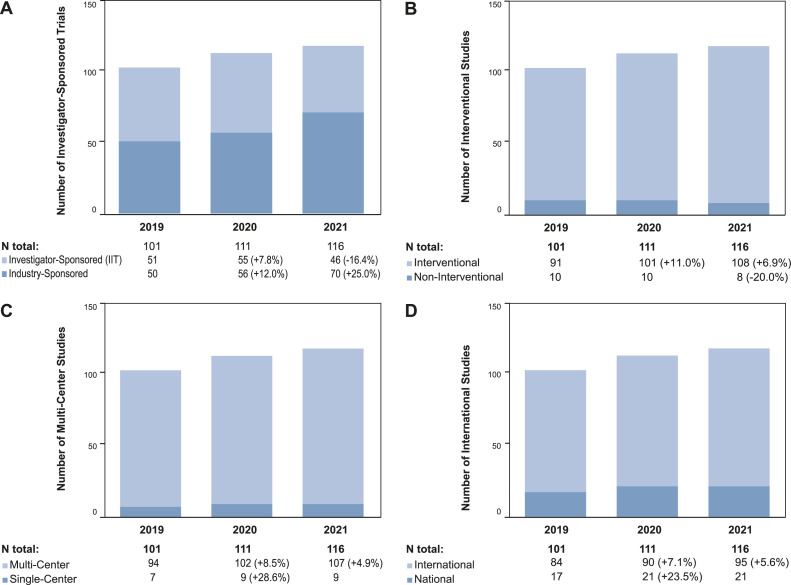
Organizational Characteristics of Registry and Phase I-III Trials from 2019-2021. A: The column chart shows the numbers of investigator-sponsored and industry-sponsored trials per year. B: The column chart shows the numbers of interventional and non-interventional trials per year. C: The column chart shows the numbers of multi-center and single-center trials per year. D: The column chart shows the numbers of national and international trials per year. Abbreviation: IIT –Investigator-initiated Trial.

**Fig. 4:**
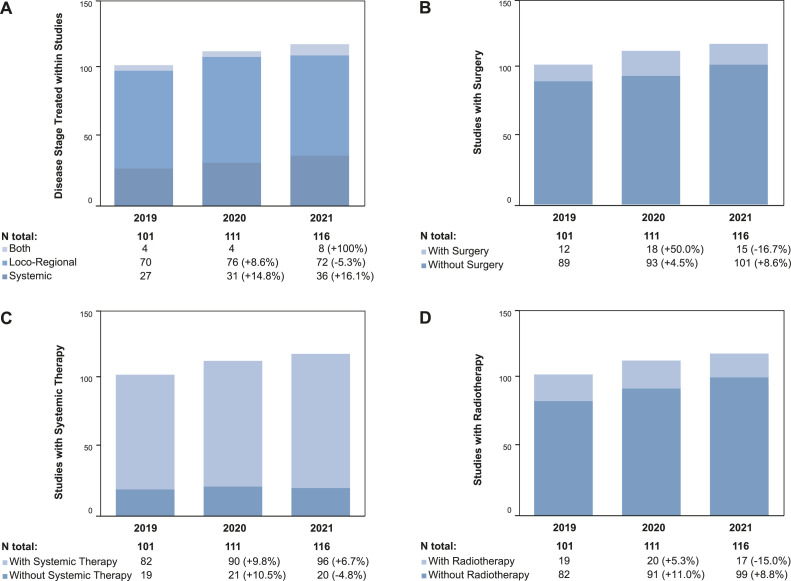
Treatment-related Characteristics of Registry and Phase I-III Trials from 2019-2021. A: The column chart shows the number of trials per year including locoregional disease, systemic disease, or both. B: The column chart shows the number of trials per year with a surgical component in the trial protocol. C: The column chart shows the number of trials per year with a systemic therapy component in the trial protocol. D: The column chart shows the number of trials per year with a radiotherapy component in the trial protocol.

In 2019, there were a total of 107 active trials including registry trials (*n*=8), clinical phase I-III trials (*n*=93), and translational studies (*n*=6) at the CCCZ, which recruited 51, 173, and 80 patients, respectively. With regard to registry and phase I-III clinical trials, 50.5 % were investigator-sponsored (*n*=51), 90.1 % (*n*=91) were interventional, 93.1 % (*n*=94) were multi-center, and 83.2 % (*n*=84) were international trials. The subject of these trial protocols were the treatment of loco-regional disease (69.3 %, *n*=70), systemic disease (26.7 %, *n*=27), or both (4.0 %, *n*=4). Trials incorporated cancer treatment by surgery (11.9 %, *n*=12), radiotherapy (18.1 %, *n*=19), and systemic treatment (81.2 %, *n*=82) (Fig. 1, Fig. 2, Fig. 3, Fig. 4).

### Changes in trials and patients numbers in 2020 and 2021

In 2020, there were a total of 120 active trials increasing to a total of 125 in 2021. The number of registry trials was nine in both years. One-hundred-three and 107 clinical phase I-III trials, as well as eight and nine translational studies recruited patients in 2020 and 2021, respectively. The numbers of recruited patients decreased for phase I-III trials (-2.3 %, *n*=169) and registry trials (-72.5 %, *n*=14) from 2019 to 2020 and increased by 10.7 % (*n*=187) and 907.1 % (*n*=141), respectively, when 2021 was compared to 2020. With regard to translational studies, recruitment numbers increased in both years by 115.0 % (*n*=172, 2020) and 96.5 % (*n*=388) in 2021. The increase from 2019 to 2021 was predominantly due to an intensified recruitment of patients into registry and translational studies. The recruitment into phase I-III clinical trials remained stable during these three years (Fig. 1, Fig. 2, Fig. 3, Supplementary Table 1).

### Changes in characteristics of registry and phase I-III trials in 2020 and 2021

During the first pandemic year, the number of investigator-sponsored (*n*=55, +7.8 %) and industry-sponsored (*n*=56, +12.0 %) trials had increased compared to 2019. The number of investigator-sponsored trials then decreased in 2021 (*n*=46, -16.4 %), while the latter was still increasing to *n*=70 (+25.0 %). Non-interventional (2020: *n*=10, +0 %; 2021: *n*=8, -20.0 %), single-center (2020: *n*=9, +28.6 %; 2021: *n*=9, +0 %), and national trials (2020: *n*=21, +23.5 %; 2021: *n*=21, +0 %) remained stable in numbers from 2019 to 2021. Those of interventional (2020: *n*=101, +11.0 %; 2021: *n*=108, +6.9 %), multi-center (2020: *n*=102, +8.5 %; 2021: *n*=107, +4.9 %), and international trials (2020: *n*=90, +7.1 %; 2021: *n*=95, +5.6 %) increased during that period (Fig. 3).

In 2020, both the number of trials investigating loco-regional (*n*=76, +8.6 %) and systemic disease (*n*=31, +14.8 %) increased. The same was true for trial protocols with (*n*=18, +50.0 %) and without (*n*=93, +4.5 %) surgery, with (*n*=20, +5.3 %) or without (*n*=91, +11.0 %) radiotherapy, and with (*n*=90, +9.8 %) or without (*n*=21, +10.5 %) systemic treatment. During the second year of the pandemic, the trends were different: Less trials investigated loco-regional disease (*n*=72, -5.3 %) while the number for trials assessing systemic disease further increased (*n*=36, +16.1 %). Also, more protocols utilized systemic treatment (*n*=96, +6.7 %), while protocol numbers incorporating surgery (*n*=15, -16.7 %) and radiotherapy (*n*=17, -15.0 %) decreased (Fig. 4).

### Assessment of associations of trial characteristics and recruiting CCCZ subunits with changes in patient numbers during the pandemic

Within the CCCZ, there were no significant differences between the subunits/clinics in changes of patient recruitment in clinical phase I-III trials when the year prior to the COVID-19 pandemic (2019) was compared to the first year of the pandemic (2020) and to 2020/2021 (Supplementary Tables 2 and 3).

Further, none of the trial characteristics mentioned above were associated with a decrease in patient recruitment in uni- and multivariate analysis when comparing trial recruitment numbers for clinical phase I-III trials from prior to the COVID-19 pandemic (2019) with the years 2020 and 2020/2021 (Table 1, Supplementary Table 4).

**Table 1:** Univariate and Multivariate Cox Regression Analysis of Trial Characteristics and Association of Changes in Trial Recruitment.

Endpoint	Successful Recruitment during COVID-19 Pandemic HR (95 % CI)			
Variable	UVA	*p*-value	MVA	p-value
Therapy				
Systemic	Reference	-	Reference	-
Local	0.98 (0.47 - 2.03)	0.996	0.95 (0.47 - 2.03)	0.892
Both	<0.01 (0.00 - Inf.)	0.996	<0.01 (<0.01 - >10)	0.996
Sugery±				
No	Reference	-	Reference	-
Yes	1.25 (0.48 – 3.22)	0.996	1.89 (0.65 – 5.50)	0.243
Radiooncology±				
No	Reference	-	Reference	-
Yes	0.50 (0.18 – 1.41)	0.810	0.33 (0.09 – 1.30)	0.104
Systemic Therapy±				
No	Reference	-	Reference	-
Yes	1.12 (0.40 – 3.17)	0.996	0.75 (0.21 – 2.70)	0.655
Trial Phases				
I	Reference	-	Reference	-
I-II	1.17 (0.34 – 4.01)	0. 996	1.17 (0.34 – 4.01)	0.799
II	0.33 (0.10 – 1.15)	0.810	0.34 (0.10 – 1.10)	0.082
II-III	0.83 (0.09 – 7.41)	0. 996	0.83 (0.09 – 7.41)	0.866
III	0.83 (0.28 – 2.48)	0. 996	0.83 (0.28 – 2.48)	0.738
Trial Design				
Non-Interventional	Reference	-	Reference	-
Interventional	0.82 (0.11 – 5.99)	0. 996	1.00 (0.13 – 7.60)	0.673
Sponsor				
Industry	Reference	-	Reference	-
IIT	0.81 (0.41 – 1.58)	0. 996	0.72 (0.34 – 1.50)	0.369
Character				
National	Reference	-	Reference	-
International	0.80 (0.31 – 2.06)	0. 996	1.18 (0.27 – 5.10)	0.825
Character				
Single-Center	Reference	-	Reference	-
Multi-Center	0.42 (0.13 – 1.40)	0.810	0.30 (0.05 – 1.80)	0.191

Abbreviations: UVA – Univariate Analysis; MVA – Multivariate Analysis; HR – Hazard Ratio; CI – Confidence Interval; IIT – Investigator-initiated Trial. For Analysis, changes in patient recruitment numbers were displayed as categorical variables. A decrease in patient recruitment from 2019 to 2020/2021 was considered as an event whereas the endpoint was defined as constancy/increase in recruitment from 2019 to 2020/2021. Correction for multiple testing was conducted using Benjamini-Hochberg procedure. For trials with recruitment in the years 2020 and 2021, the mean value was used for analysis.

± Character as component of the trial protocol.

## Discussion

The healthcare systems around the world have experienced significant disruption due to the COVID-19 pandemic, also widely affecting the implementation, execution and recruitment of clinical trials. However, actual data describing clinical trial activities during the COVID-19 pandemic are rare. Here, we present the development of clinical trial activities and patient recruitment into clinical studies of solid malignancies during the COVID-19 pandemic in a tertiary cancer center.

In contrast to most international reports, our analysis revealed increasing numbers of active clinical trials and patient enrolment numbers during the COVID-19 pandemic. At our tertiary cancer center, the number of actively recruiting trials increased in both pandemic years 2020 and 2021 and the number of patients enrolled into phase I-III clinical trials remained stable. In line with our findings, two large US cancer centers reported an increase in clinical trials and patient enrolment in early 2021 compared to the pre-pandemic period [18]. Since our data were only provided annually, probable interim decreases in numbers might have been missed, especially during the first wave of the pandemic. Furthermore, an analysis of the number of clinical trials stopped owing to COVID-19 on ClinicalTrials.gov showed that following an initial peak in May/June 2020, the number of monthly stopped clinical trials dropped again. The impact of COVID-19 on oncology trials was less severe compared to non-oncology trials [19].

One possible explanation for the lack of a negative impact of the pandemic on our center's clinical trial activities could be the less stringent COVID-19 policies in Switzerland compared to other countries during the pandemic. This also reflects a less profound impact of the pandemic on the awareness and behavior of the Swiss population. Hale and colleagues assessed COVID-19 measures and developed composite indexes indicating the rigor of different or combined pandemic containment measures by countries. If policies varied at the subnational level, the index always incorporated the level of the strictest sub-region on a time scale. The analysis showed that Swiss COVID-19 policies were more moderate than for example in Germany or the USA [11,12]. It appears that these less stringent national and regional COVID-19 restrictions were translated into successful continuation of non-elective oncological health care, especially oncological surgery, and successful continuation of oncological clinical trial activities [13].

Globally, the impact of the COVID-19 pandemic generally reduced the initiation of clinical trials. Monthly trial activation numbers dropped by up to 57 % in the first year of the pandemic [6,[20], [21], [22]]. This development was also reported for cancer clinical trials. Several studies showed that trial initiations and enrolment were severely reduced during the first pandemic year [5,7,23,24]. A survey among US cancer patients indicated that the fear of exposure to COVID-19 reduced the likelihood to participate in clinical trials whereas two surveys among physicians revealed challenges with patient enrolment, protocol adherence due to decreased patient visits, milestone delays, and reduced staff and resources [25], [26], [27].

To the best of our knowledge, only a single tertiary cancer center reported on the impact of COVID-19 with regard to their patient recruitment numbers into clinical trials so far. McLaughlin et al. reported a 33 % reduction of screened patients and a 60 % reduction of enrolled patients when the first half of 2020 was compared to the first half of 2019 at an Irish cancer center [28]. An analysis of 64 National Cancer Institute-Designated Cancer Centers (NCI-DCC) showed a decline of 13.7 % in the enrolment of industry-sponsored trials, of 18.1 % for institutional-, and of 30.5 % for third-party funded trials when the year of 2020 was compared to 2019. During that time, the numbers of active trials remained unchanged. Interestingly, the number of trials supported by the NCI or other national institutes and their enrolment remained stable. However, the total number of actively recruiting trials and their recruitment numbers dropped by approximately 20 and 50 % when the first half of 2021 was compared to the year of 2020, respectively. The numbers show a delayed reduction in cancer clinical trials following the onset of the pandemic [29]. In addition to the direct impact of the pandemic on general patient care, there were also fewer initial cancer diagnoses at the onset of the pandemic [30,31]. During that time, the American Society of Clinical Oncology (ASCO) recommended to reduce patient contact with health care facilities and therefore to postpone cancer screening procedures such as mammographies and colonoscopies [32,33]. In that context, a cross-sectional study revealed a significant decline in the number of first diagnoses of solid cancer in the USA during the first months of COVID-19 [33]. Corresponding results were published for the Netherlands and the United Kingdom [34]. The authors predicted that delays in cancer diagnoses would be associated with poorer clinical outcome [33]. Indeed, several studies showed that more patients were subsequently diagnosed with more advanced cancer stages [35], [36], [37]. As a result, recruitment into early-stage cancer trials could have been impaired for that reason.

Several authors have addressed the question of how clinical trials can be successfully conducted during the pandemic. Coherently, study decentralization via telemedicine and remote study visits were suggested. Furthermore, protocols should be reviewed for dispensable assessments and procedures. Where possible, the delivery of investigational products directly to patient can be considered [8,9,26,38,39,10].

Trial and recruitment numbers were only available annually, which does not allow a more detailed analysis of clinical trial activities during the pandemic waves; such intermittent effects were certainly evident at our cancer center, however, were compensated for by rapid re-initiation of clinical trial activities and potentially increased activities after the waves. This indicates that short-term disturbances of clinical trial activities can be rather well compensated.

## Conclusion

The healthcare systems around the world have experienced significant disruption due to the COVID-19 pandemic also widely affecting the implementation, execution, and recruitment of clinical trials. Adapted workflows and study decentralization via telemedicine and remote study visits were suggested to adapt to this challenging situation. Data from our tertiary cancer center show that trial activities were maintained at a high level during the pandemic, indicating that less stringent COVID-19 policies in Switzerland compared to other countries were successfully translated into the continuation of oncological clinical trial activities.

## CRediT authorship contribution statement

**Jens von der Grün:** Conceptualization, Formal analysis, Data curation, Writing – original draft, Writing – review & editing, Visualization. **Maiwand Ahmadsei:** Formal analysis, Data curation, Writing – review & editing, Visualization. **Isabel Breyer:** Conceptualization, Methodology, Investigation, Formal analysis, Writing – review & editing. **Christian Britschgi:** Investigation, Writing – review & editing. **Daniel Eberli:** Investigation, Writing – review & editing. **Thomas Hermanns:** Investigation, Writing – review & editing. **Joanna Mangana:** Methodology, Investigation, Writing – review & editing. **Henrik Petrowsky:** Investigation, Writing – review & editing. **Egle Ramelyte:** Investigation, Writing – review & editing. **Patrick Roth:** Investigation, Writing – review & editing. **Gabriel Schär:** Investigation, Writing – review & editing. **Isabelle Opitz:** Investigation, Writing – review & editing. **Michael Weller:** Investigation, Writing – review & editing. **Andreas Wicki:** Investigation, Writing – review & editing. **Isabell Witzel:** Investigation, Writing – review & editing. **Panagiotis Balermpas:** Investigation, Writing – review & editing. **Matthias Guckenberger:** Conceptualization, Methodology, Formal analysis, Data curation, Writing – original draft, Writing – review & editing, Project administration.

## Declaration of Competing Interest

The authors declare the following financial interests/personal relationships which may be considered as potential competing interests: CB reported consulting or advisory roles for AstraZeneca, Pfizer, Roche, Takeda, Janssen-Cilag, Boehringer Ingelheim, Merck KGaA, and Sanofi; research funding from Bayer; and travel accommodations and expenses from AstraZeneca, Takeda and Roche; all over the last five years. MW has received research grants from Quercis and Versameb, and honoraria for lectures or advisory board participation or consulting from Bayer, Curevac, Medac, Novartis, Novocure, Orbus, Philogen, Roche and Sandoz. IO reported the following disclosures: Roche (Institutional Grant and Speakers Bureau), AstraZeneca (Advisory Board and Speakers Bureau), MSD (Advisory Board), BMS (Advisory Board), Medtronic (Institutional Grant), Intuitive (Proctorship). PR has received honoraria for lectures or advisory board participation from Alexion, Bristol-Myers Squibb, Boehringer Ingelheim, Debiopharm, Midatech Pharma, Novocure, QED, and Roche and research support from Merck Sharp and Dohme and Novocure. JM has intermittent project focused consultant or advisory relationships with Merck/Pfizer, Merck Sharp & Dohme, Amgen, Novartis, Roche, Bristol Myers Squibb and Pierre Fabre and has received travel support from Ultrasun, L’ Oreal, Merck Sharp & Dohme, Bristol Myers and Squibb und Pierre Fabre outside of the submitted work. ER received research funding by Amgen; consulting or advisory roles for Sanofi/Regeneron and Amgen; honoraria from Pierre Fabre, Lilly, Galderma, MSD, Sanofi, and BMS GmbH & Co KG; Travel expenses from Amgen and Sanofi. All other authors reported no potential conflicts of interest

## Appendix Supplementary materials

Image, application 1

Image, application 2

Image, application 3
