# Supplementary material for: Patient recruitment into clinical studies of solid malignancies during the COVID-19 pandemic in a tertiary cancer center
Source: Neoplasia. 2023 Oct 27;46:100946. doi: 10.1016/j.neo.2023.100946 (PMC10630114; doi:10.1016/j.neo.2023.100946)
Supplement: Supplementary file 1 [file mmc1.docx]

**Supplementary Table 1: Patient Recruitment between 2019 and 2021.** Abbreviation: CCCZ - Comprehensive Cancer Center Zurich.

| Characteristics per  CCCZ Subunit | Year 2019, N | Year 2020, N (%) | Year 2021, N (%) |
| --- | --- | --- | --- |
| Head and Neck Oncology |  |  |  |
| Translational Studies | 0 | 31 | 26 |
| Registry Trials | 0 | 0 | 0 |
| Phase I-III Trials (total) | 3 | 10 | 13 |
| Phase I Trials | 0 | 0 | 0 |
| Phase I/II Trials | 0 | 0 | 0 |
| Phase II Trials | 3 | 9 | 13 |
| Phase II/III Trials | 0 | 0 | 0 |
| Phase III Trials | 0 | 1 | 0 |
| Total No. of Patients | 3 | 41 | 39 |
| Total No. of Studies | 4 | 6 | 6 |
| Dermatological Oncology |  |  |  |
| Translational Studies | 0 | 0 | 0 |
| Registry Trials | 4 | 4 | 1 |
| Phase I-III Trials (total) | 60 | 66 | 60 |
| Phase I Trials | 16 | 6 | 7 |
| Phase I/II Trials | 3 | 33 | 25 |
| Phase II Trials | 28 | 15 | 20 |
| Phase II/III Trials | 0 | 1 | 2 |
| Phase III Trials | 13 | 11 | 6 |
| Total No. of Patients | 64 | 70 | 61 |
| Total No. of Studies | 22 | 25 | 36 |
| Neuro-Oncology |  |  |  |
| Translational Studies | 0 | 0 | 0 |
| Registry Trials | 0 | 0 | 0 |
| Phase I-III Trials (total) | 30 | 26 | 44 |
| Phase I Trials | 6 | 1 | 2 |
| Phase I/II Trials | 8 | 10 | 16 |
| Phase II Trials | 1 | 13 | 26 |
| Phase II/III Trials | 0 | 0 | 0 |
| Phase III Trials | 15 | 2 | 0 |
| Total No. of Patients | 30 | 26 | 44 |
| Total No. of Studies | 9 | 14 | 11 |
| Sarcoma Oncology |  |  |  |
| Translational Studies | 0 | 0 | 0 |
| Registry Trials | 0 | 0 | 0 |
| Phase I-III Trials (total) | 5 | 6 | 1 |
| Phase I Trials | 0 | 0 | 0 |
| Phase I/II Trials | 0 | 0 | 0 |
| Phase II Trials | 5 | 5 | 1 |
| Phase II/III Trials | 0 | 0 | 0 |
| Phase III Trials | 0 | 1 | 0 |
| Total No. of Patients | 5 | 6 | 1 |
| Total No. of Studies | 5 | 4 | 3 |
| Thoracic Oncology |  |  |  |
| Translational Studies | 0 | 52 | 108 |
| Registry Trials | 0 | 0 | 0 |
| Phase I-III Trials (total) | 16 | 6 | 17 |
| Phase I Trials | 0 | 0 | 0 |
| Phase I/II Trials | 7 | 0 | 0 |
| Phase II Trials | 3 | 3 | 16 |
| Phase II/III Trials | 0 | 0 | 0 |
| Phase III Trials | 6 | 3 | 1 |
| Total No. of Patients | 16 | 58 | 125 |
| Total No. of Studies | 19 | 16 | 16 |
| Urogenital Oncology |  |  |  |
| Translational Studies | 142 | 121 | 284 |
| Registry Trials | 79 | 86 | 154 |
| Phase I-III Trials (total) | 43 | 7 | 99 |
| Phase I Trials | 0 | 0 | 0 |
| Phase I/II Trials | 0 | 2 | 0 |
| Phase II Trials | 9 | 10 | 16 |
| Phase II/III Trials | 6 | 8 | 2 |
| Phase III Trials | 5 | 8 | 13 |
| Total No. of Patients | 20 | 28 | 31 |
| Total No. of Studies | 13 | 18 | 15 |
| Visceral Oncology |  |  |  |
| Translational Studies | 0 | 0 | 47 |
| Registry Trials | 0 | 0 | 0 |
| Phase I-III Trials (total) | 8 | 12 | 18 |
| Phase I Trials | 0 | 3 | 1 |
| Phase I/II Trials | 1 | 0 | 1 |
| Phase II Trials | 3 | 5 | 12 |
| Phase II/III Trials | 0 | 0 | 0 |
| Phase III Trials | 4 | 4 | 4 |
| Total No. of Patients | 8 | 12 | 65 |
| Total No. of Studies | 13 | 16 | 21 |
| Gynecological Oncology |  |  |  |
| Translational Studies | 1 | 3 | 3 |
| Registry Trials | 4 | 3 | 41 |
| Phase I-III Trials (total) | 15 | 14 | 0 |
| Phase I Trials | 0 | 0 | 0 |
| Phase I/II Trials | 0 | 0 | 0 |
| Phase II Trials | 0 | 0 | 0 |
| Phase II/III Trials | 0 | 0 | 0 |
| Phase III Trials | 15 | 14 | 0 |
| Total No. of Patients | 20 | 20 | 44 |
| Total No. of Studies | 18 | 17 | 13 |
| Disease Agnostic Systemic Oncology |  |  |  |
| Translational Studies | 0 | 0 | 0 |
| Registry Trials | 0 | 0 | 0 |
| Phase I-III Trials (total) | 16 | 1 | 3 |
| Phase I Trials | 0 | 0 | 0 |
| Phase I/II Trials | 0 | 0 | 0 |
| Phase II Trials | 0 | 0 | 0 |
| Phase II/III Trials | 16 | 1 | 3 |
| Phase III Trials | 0 | 0 | 0 |
| Total No. of Patients | 16 | 1 | 3 |
| Total No. of Studies | 4 | 4 | 4 |

**Supplementary Table 2: Changes in Patient Numbers in the Recruitment of Phase I-III Clinical Trials from 2019 to 2020.** Abbreviation: CCCZ - Comprehensive Cancer Center Zurich; *Correction for multiple testing was conducted using Benjamini-Hochberg procedure. ^#^Total number of phase I-III clinical trials with recruitment Activity in 2019 and 2020, N=82.

| CCCZ Subunit | Trials^#^ with Decrease in Recruitment, N (%) | Trials^#^ with Constancy/Increase in Recruitment, N (%) | p-value* |
| --- | --- | --- | --- |
| Head and Neck Oncology | 0 (0.0%) | 3 (100.0%) |  |
| Dermatological Oncology | 7 (43.8%) | 9 (56.3%) |  |
| Neuro-Oncology | 5 (62.5%) | 3 (37.5%) |  |
| Sarcoma Oncology | 1 (25.0%) | 3 (75.0%) |  |
| Thoracic Oncology | 5 (37.5%) | 9 (64.3%) |  |
| Urogenital Oncology | 3 (33.3%) | 6 (66.7 %) |  |
| Visceral Oncology | 2 (15.4%) | 11 (84.6%) |  |
| Gynecological Oncology | 3 (27.3%) | 8 (72.7%) |  |
| Disease Agnostic Systemic Oncology | 2 (50.0%) | 2 (50.0%) | 0.822 |

**Supplementary Table 3: Changes in Patient Numbers in the Recruitment of Phase I-III Clinical Trials from 2019 to 2020/2021.** Abbreviation: CCCZ - Comprehensive Cancer Center Zurich; *Correction for multiple testing was conducted using Benjamini-Hochberg procedure. ^#^Total number of phase I-III clinical Trials with recruitment activity in 2019 and/or 2020/2021, N=82; ^¥^For trials with recruitment in the years 2020 and 2021, the mean value was used for analysis.

| CCCZ Subunit | Trials^#^ with Decrease^¥^ in Recruitment, N (%) | Trials^#^ with Constancy/Increase^¥^ in Recruitment, N (%) | p-value* |
| --- | --- | --- | --- |
| Head and Neck Oncology | 0 (0.0%) | 3 (100.0%) |  |
| Dermatological Oncology | 10 (62.5%) | 6 (37.5%) |  |
| Neuro-Oncology | 6 (75.0%) | 2 (25.0%) |  |
| Sarcoma Oncology | 1 (25.0%) | 3 (75.0%) |  |
| Thoracic Oncology | 5 (35.7%) | 9 (64.3%) |  |
| Urogenital Oncology | 3 (33.3%) | 6 (66.7%) |  |
| Visceral Oncology | 2 (15.4%) | 11 (84.6%) |  |
| Gynecological Oncology | 6 (54.5%) | 5 (45.5%) |  |
| Disease Agnostic Systemic Oncology | 2 (50.0%) | 2 (50.0%) | 0.470 |

**Supplementary Table 4: Correlation of Changes in Patient Recruitment and Characteristics of Phase I-III Clinical Trials.** Abbreviation: IIT – Investigator-initiated Trial; *Correction for multiple testing was conducted using Benjamini-Hochberg procedure. ^#^Decrease vs. Constrancy/Increase; Total number of phase I-III clinical Trials with recruitment activity in 2019 and/or 2020/2021, N=82; ^¥^For trials with recruitment in the years 2020 and 2021, the mean value was used for analysis; ^±^Character as component of the trial protocol.

| Character | Changes^#^ from 2019 to 2020, p-value* | Changes^#^ from 2019 to 2020/2021^¥^, p-value* |
| --- | --- | --- |
| Therapy: Local vs. Systemic vs. Both | 0.822 | 0.610 |
| Sugery vs. No Surgery^±^ | 0.846 | 0.804 |
| Radiooncology vs. No Radiooncology | 0.822 | 0.470 |
| Systemic Therapy vs. No Systemic Therapy | 0.822 | 0.844 |
| Trial Phases: I vs. I-II vs. II vs. II-III vs. III | 0.822 | 0.844 |
| Interventional Trial vs. Non-Interventional Trial | 0.846 | 0.804 |
| Sponsor: Industry vs. IIT | 0.882 | 0.804 |
| National vs. International | 0.853 | 0.470 |
| Single-Center vs. Multi-Center | 0.822 | 0.470 |
