# Supplementary figures and images for: Patient recruitment into clinical studies of solid malignancies during the COVID-19 pandemic in a tertiary cancer center
Source: Neoplasia. 2023 Oct 27;46:100946. doi: 10.1016/j.neo.2023.100946 (PMC10630114; doi:10.1016/j.neo.2023.100946)

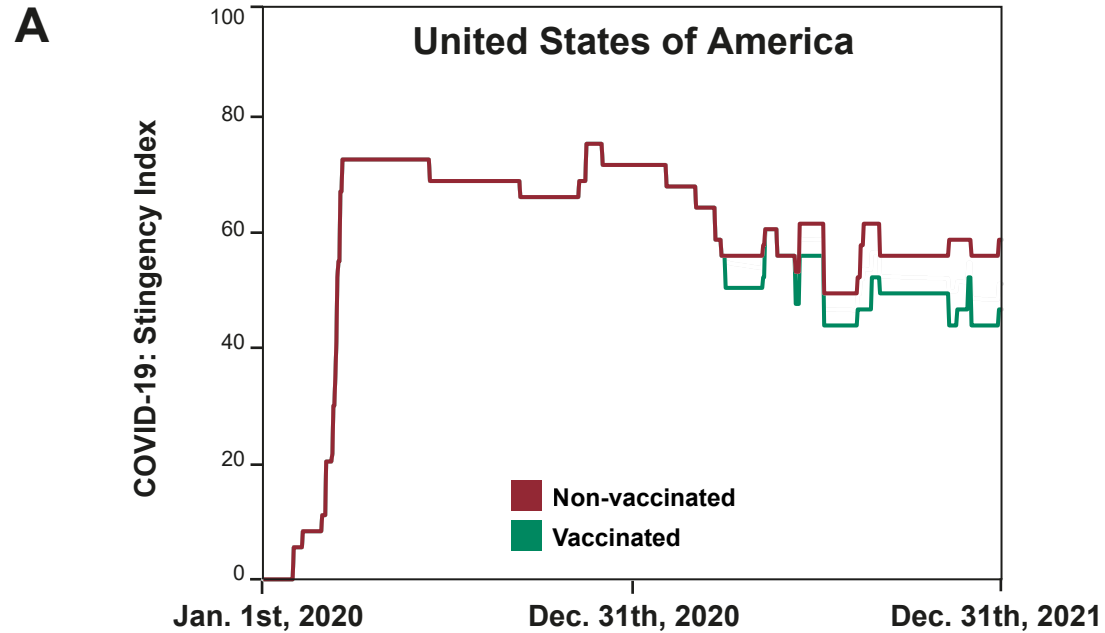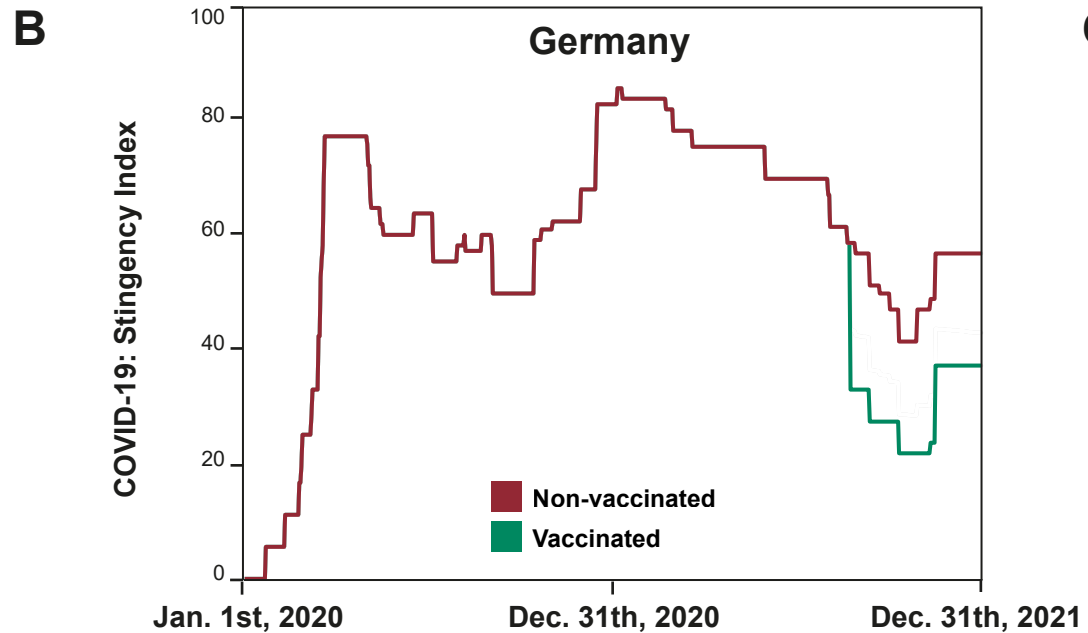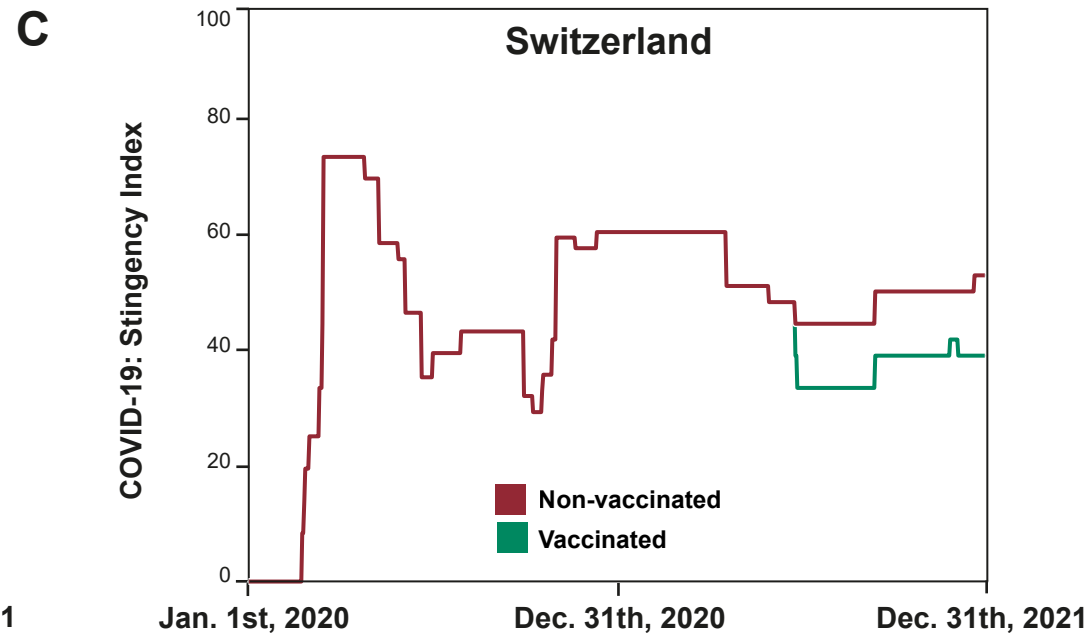

Supplement: Supplementary file 2 [file mmc2.pdf]

# COVID-19: Containment and Health Index

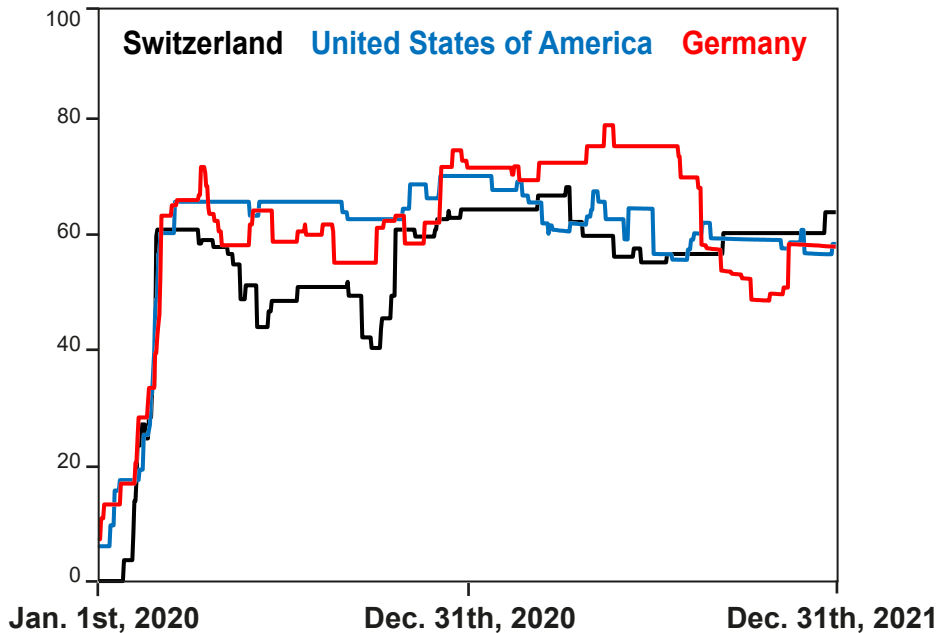

Supplement: Supplementary file 3 [file mmc3.pdf]
